# Anticipating Unipolar Depression and Bipolar Depression in young adult with first episode of depression using childhood trauma and personality

**DOI:** 10.3389/fpubh.2022.1061894

**Published:** 2023-01-10

**Authors:** Jiali Liu, Yuanyuan Wang, Amanda Wilson, Hui Chen, Peiqu Liu, Xianliang Chen, Huajia Tang, Chenyuli Luo, Yusheng Tian, Xiaoping Wang, Xia Cao, Jiansong Zhou

**Affiliations:** ^1^National Clinical Research Center for Mental Disorders, Department of Psychiatry, The Second Xiangya Hospital of Central South University, Changsha, Hunan, China; ^2^Division of Psychology, Faculty of Health and Life Sciences, De Montfort University, Leicester, United Kingdom; ^3^Dongguan Mental Health Center, Dongguan, Guangdong, China; ^4^Health Management Center, Health Management Research Center of Central South University, The Third Xiangya Hospital, Central South University, Changsha, Hunan, China

**Keywords:** childhood trauma, Eysenck Personality Inventory, depressive episode, young adult, Bipolar Depression

## Abstract

**Objective:**

Relevant research focusing on young adults with Unipolar Depression (UD) and Bipolar Depression (BD) is limited. The current research aims to investigate childhood trauma and personality traits in young adults with UD and BD.

**Methods:**

Two hundred and thirty-five patients in a first depressive episode (diagnosed UD and BD), 16–25 years old, were recruited from Second Xiangya Hospital. And 79 healthy controls (HC) were recruited from the community to form the comparison group. Patients' childhood trauma was measured by the Childhood Trauma Questionnaire (CTQ), and personality was measured by Eysenck Personality Inventory (EPI). The Kruskal-Wallis test was used to compare depression, anxiety, CTQ, and EPI scores between the HC (*n* = 79), UD (*n* = 131), and BD (*n* = 104) groups. Factors independently associated with mood disorders and BD were determined using binary logistic regression analyses.

**Results:**

Compared with HC, mood disorders had more severe anxiety and depression symptoms, and higher CTQ. Emotional abuse (OR = 1.47; 95% CI = 1.08–2.01), emotional neglect (OR = 1.24; 95% CI = 1.05–1.46), and neuroticism (OR = 1.25; 95% CI = 1.16–1.35) were associated with significantly increased odds of mood disorders. Whereas, higher extraversion scores were a protective factor for mood disorders. Compared with UD, BD had more severe anxiety symptoms, and higher CTQ, than extraversion and neuroticism personality scores. Anxiety (OR = 1.06; 95% CI = 1.02–1.08) and extraversion (OR = 1.05; 95% CI = 1.03–1.09) were associated with significantly increased odds of BD.

**Conclusion:**

Interventions to prevent childhood trauma may improve young adults' mental health. Using childhood trauma and personality to anticipate BD and UD creates more accurate treatment for young adults with first depression.

## 1. Introduction

In 2022, the World Health Organization (WHO) estimates that there are 280 million people with depression in the world, accounting for 3.8% of the total population and resulting in a high global burden, particularly in low and middle-income countries where 75% of the population receive no treatment (1). Based on past epidemiological trends, the prevalence of mood disorders among youth has increased. In particular, since the pandemic began, there has been more than a 2-fold increase (from 8.5 to 28%) in youth presenting with clinically significant depression (2). Furthermore, depression is one of the main causes of illness and disability globally among young adults (1). More seriously, experiencing depression often leads to a decline in patients' quality of life, an increase in death rates, and a decline in cognitive function, including: continuous attention, verbal memory, the ability to change settings, and inhibition, and control (3). The diagnosis of Bipolar Depression (BD) can be highly unpredictable and convoluted, BD can be misdiagnosed as other clinically significant depressive symptoms, which may indicate a diagnosis of Unipolar Depression (UD) or Major Depressive Disorder (MDD) (4). Indeed, the symptomatic overlap of depressive episodes between BD and UD is widely considered a diagnostic and therapeutic problem. Mania remains the only dependable distinguishing marker (5). This study intends to distinguish UD symptoms from BD symptoms using measures of childhood trauma and personality traits.

As stated, BD and UD are prevalent in young adult, especially in the case of childhood trauma. In a multivariate model of the pathogenesis of depression, childhood trauma is a common psycho-social factor. Specifically, it is closely related to chronic depression with a long duration of the illness and higher severity of symptoms. Childhood trauma usually refers to adverse events that occurred in childhood, including abuse and neglect of physical and mental health (6). According to a WHO survey, more than one-third of the world's population has experienced childhood trauma (7), meaning they are highly susceptibility to developing BD and UD. Epidemiological data in China shows that incidences of physical abuse, sexual abuse, neglect, and emotional abuse in childhood are 32.40–67.30%, 10.20–25.50%, 22.40–54.90%, and 10.60–67.10%, respectively (8). Childhood trauma can lead to an immediate or lasting adverse effects on physical and mental health and may account for a third of all psychiatric disorders (7). Using a random-effects meta-analysis, the researchers found that individuals with childhood trauma were more likely to develop depression than individuals without (9). Childhood trauma is also considered to be related to high comorbidity and severity of BD, and childhood trauma rates are reportedly as high as 49% in BD samples (10), suggesting that BD is under-reported in the WHO data. Numerous studies have observed that childhood trauma can have a lasting effect on individuals, it has been demonstrated there is a negative impact where having a history of childhood sexual abuse can adversely affect adult outcomes, such as a likelihood of developing BD (11). Furthermore, a study on the elderly showed that childhood trauma increased the risk of UD later in life (12). There also have been studies indicating that childhood trauma is related to personality trait. This is supported in findings among Chinese young adults, where a significant positive correlation has been found between childhood trauma total scores, as well as the subscales of emotional abuse, sexual abuse, Eysenck Personality Inventory-neuroticism, and psychoticism scores (13). Differences have further been found between personality and mood disorders when using a healthy control group. Both BD and UD patients reportedly score significantly higher on the neuroticism scale and lower on the extraversion scale than the general population; however other studies have reported inconsistent findings. When compared to UD patients, BD patients have lower, higher, or equal neuroticism scores, and higher or equal extraversion scores (14). The reason for this disunity may be that personality traits have a certain degree of heredity, as supported by a twin studies (15). Therefore, it is important to compare UD and BD from the perspective of childhood trauma and personality traits to better understand UD and BD when compared to a HC.

Previous studies have shown that severe depression, atypical symptoms of depression, parental history of BD, comorbid psychotic symptoms, and childhood trauma are all risk factors for UD developing into BD (16). The etiology of UD and BD may involve both environmental and genetic factors. Powerful associations between childhood traumatic experiences and the development of personality traits and depression have been well-documented, suggesting that childhood trauma may increase vulnerability for both disorders. Furthermore, evidence supports a dose-response relationship between trauma and other disorders, along with mood disorders, where the strongest associations have been found between emotional abuse and neglect (17). Compared with UD, childhood trauma also appears to be associated with BD. The rate of experiencing childhood trauma is 55.5% in patients with UD, with the rate being up to 61.8% in BD patients (18). These temperament differences play an important role in distinguishing BD from UD, and a limited number of studies have explored the correlative differences between childhood trauma and personality in BD and UD.

In China, the lifetime prevalence rate of depression is 6.9%, and more than 95 million people suffer from depression, accounting for 7.3% of the total population, which is the largest number of depressed patients in the world (19). Therefore, the present study anticipates BD and UD in young adult with mood disorder-based depression and childhood trauma. It first aims to explore the differences of childhood trauma and personality traits between BD, UD, and the control group. Secondly, it aims to evaluate the relationship between childhood trauma and personality among UD and BD, and identify risk factors and protective factors associated with UD and BD among young adults.

## 2. Materials and methods

### 2.1. Participants and study design

**Figure 1:**
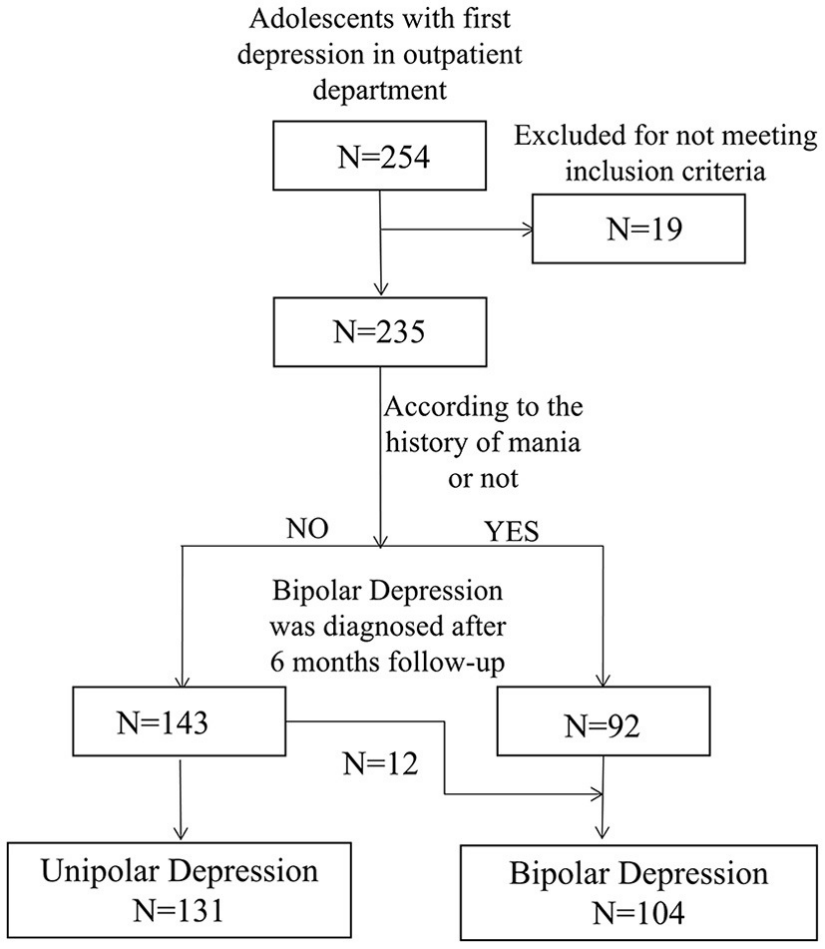
Flow chart of study participants.

This study is part of the Youth Depression Cohort (Second Xiangya Hospital) study (YDC-XY), which was conducted in the Second Xiangya Hospital of Central South University from January 1st, 2018 to June 2nd, 2021. A flowchart describing the patient recruitment procedure is presented in Figure 1. Participants were consecutively recruited, and inclusion criteria was as follows: aged 16–25 years, in a current and first depressive episode, no treatment with prescription medication, diagnosed as having a mood disorder according to DSM-5 (The Diagnostic and Statistical Manual of Mental Disorders), a score ≥7 points on the Beck Depression Inventory (BDI), and no psychiatric treatment during the past 3 months. Exclusion criteria was as follows: having comorbidity with neurological conditions, having a severe somatic disease, and/or substance use disorder. Informed consent was obtained from all participants before commencing the study (minors provided assent and informed consent from their guardians by signing an informed consent form). The study approval was granted by the Ethics Committee of the Second Xiangya Hospital of Central South University (Approval No. 2018-007) on December 27th, 2018.

A total of 235 young adults in a current depressive episode (58 males and 177 females, mean age: 19.6 ± 2.6) who met the eligibility criteria were included in this study. Then, outpatients were divided into two groups (1) UD group and (2) BD group, using the diagnostic criteria according to the Diagnostic and Statistical Manual of Mental Disorders-5 (DSM-5). Among the 235 outpatients, 131 (55.7%) patients were diagnosed with UD (38 males and 93 females, mean age: 19.6 ± 2.4) and 104 (44.2%) with BD (20 males and 84 females, mean age: 19.6 ± 2.7). Meanwhile, 79 HC individuals (24 males and 55 females, mean age: 19.6 ± 2.6) were recruited and matched by age, sex, and years of education.

### 2.2. Assessments

A face-to-face interview confirmed a psychiatric diagnosis of UD or BD, which was completed by a senior psychiatrist using the Mini-International Neuropsychiatric Interview (M.I.N.I). In addition, a short structured psychiatric diagnostic interview was designed to meet the need for a quick but accurate structured interview procedure. Socio-demographics and clinical data were collected including age, sex, educational level (years of schooling), marital status (divided into single, in a relationship, married, unmarried and other), nationality (all Han Chinese), and occupation (divided into students, suspended, working, and other). A history of smoking, drinking, and drug use, as well as a family history of psychiatric disorders, were also captured.

Beck Depression Inventory (BDI), is a self-rated depression scale. There are many versions, in this study the shortened Beck Depression Inventory (BDI-13) (20) was used to assess the severity of depressive symptoms. Studies have indicated that the BDI-13 has a high degree of internal consistency, correlates well with the clinician's ratings of depression, and shows a high correlation with the original 21-item form. Each item is rated on a Likert scale from “0 (not at all)” to “3 (severely)” for depression, pessimism, sense of failure, lack of satisfaction, self-guilt, self-disappointment, negative tendency, social withdrawal, indecision, self-image change, work difficulties, fatigue, and loss of appetite. The total score of the scale ranges from 0 to 39 with a higher score indicating more severe depressive symptoms. Wenhui et al. have shown that the Chinese version has good reliability and validity in screening depression among young adults in China (21). In this study, depression scores above mild depression (BDI-13 total score > 7) were considered as the depression cut-off value. The BDI-13 has good internal reliability with Cronbach's α 0.93 in the young adult samples.

Beck Anxiety Inventory (BAI) (22) was used to assess the severity of anxiety symptoms. It is a self-rating scale consisting of 21 four-point items with each item rated from “0 (not at all)” to “3 (severely)” for anxiety and the total score of the scale ranges from 0 to 63 with a higher total score indicating more severe anxiety symptoms. The Chinese version of BAI was found to have satisfactory validity and reliability in both young adults and adults (23). The BAI-21 has good internal reliability with Cronbach's α 0.94 in our young adult samples.

The Childhood Trauma Questionnaire-Short Form (CTQ-SF), compiled by Bernstein et al. (24), is a 28-item self-report instrument and one of the universally recognized tools for measuring childhood trauma. The questionnaire has five subscales: emotional abuse, emotional neglect, physical abuse, physical neglect, and sexual abuse. Each subscale is rated from “1(not at all)” to “5 (very often).” Emotional abuse refers to an atmosphere of hatred, threat, degradation, and humiliation. An example of an item from the CTQ-SF measuring emotional abuse is: “Someone at home said something that made me sad or insulted me.” Physical abuse refers to attacks that cause harm to the physical body, for example: “Someone at home beat me so hard that I had to go to the hospital.” The sexual abuse subscale refers to any unwanted sexual contact or coercion between children and between children and adults. Example items include: “Someone made me do sex-related things with him/her by threatening or cheating.” The emotional neglect subscale refers to a lack of support or emotional needs such as love and a sense of belonging. An example of an item includes: “Home is my source of strength and support.” Finally, physical neglect refers to the lack of basic physical needs, such as food, shelter, and a sense of security. Each component defined in the CTQ-SF scale manual compiled by Bernstein et al. has a threshold score of moderate and severe trauma: Emotional abuse 13 points; Physical abuse 10 points; Sexual abuse eight points; Emotional neglect 15 points; and Physical neglect is 10 points. Higher scores suggest the individual has experienced corresponding types of Childhood trauma. Studies have shown that the Chinese version of the CTQ-SF has good reliability and validity in screening for childhood trauma in China (25). The Cronbach's α coefficient of the Chinese CTQ-SF in this study is 0.61.

There are many versions of the Eysenck Personality Inventory, in this study the Chinese version of the Eysenck Personality Inventory (EPI-RSC) was used. EPI-RSC was used to measure personality traits of young adults in this study, which is based on the EPQ-R short Scale (EPQ-RS) and had satisfactory reliability and validity in Chinese youth 16 years or older (26). It is a self-rating scale consisting of 48 questions, including the four subscales mentioned. The psychoticism scale represents mental health quality. The higher the psychoticism value, the more lonely, inhumane, and hostile someone is. The extraversion scale is used to evaluate extroversion traits vs. introversion traits. The higher the extraversion score, the more impulsive and emotionally exposed the individual is. The neuroticism scale measures emotional stability; those with higher neuroticism scores are seen as being overwhelmed by various stimuli and are emotionally unstable, and the lie scale measures how much integrity an individual has where higher scores represent less integrity. The Cronbach's α coefficient of EPI-RSC in this study is 0.65.

### 2.3. Statistical analysis

All data were analyzed using SPSS 25.0. Quantitative data are expressed in the form of mean ± standardized deviation (SD). The Kruskal-Wallis test and Chi-squared tests were used to compare differences in demographic and clinical data between groups. Binary logistic regression models were executed to examine the independent variables associated with mood disorders and risk factors of BD. Variables with *P* < 0.1 in the Kruskal-Wallis test were entered into the regression model as independent variables. Moreover, the association between the subscale score of the EPI and CTQ was tested using Spearman's correlation coefficient. Significance level was set at *P* < 0.05 two-sided.

## 3. Results

The socio-demographic and clinical data of the three groups are shown in Table 1. The three groups (UD, BD, and HC) did not significantly differ in age or gender, and the sex distribution of the three groups was similar, with more females than males, and that there was no significant difference in years of education.

**Table 1:** Clinical and demographic characteristics and questionnaire measurement results of healthy controls and mood disorder patients (UD and BD).

	**HC (*n* = 79)**	**UD (*n* = 131)**	**BD (*n* = 104)**	**χ^2^/K-W**	**Sig (χ^2^/*t*/*U*)**
**Continuous variables (mean** ±**SD)**					
Age, years	19.6 ± 2.6	19.6 ± 2.4	19.6 ± 2.7	0.18	0.916
Education, years	13.8 ± 2.5	13.3 ± 2.1	13.0 ± 2.4	4.06	0.053
BDI	3.0 ± 2.5^*^	19.5 ± 5.6	21.2 ± 6.1	180	< 0.001
BAI	3.4 ± 3.3^***^	22.1 ± 10.8	27.4 ± 11.2^###^	168.71	< 0.001
**CTQ**					
Total score	31.4 ± 5.6^***^	49.6 ± 12.1	53.9 ± 15.7^#^	129.14	< 0.001
Emotional abuse	6.1 ± 1.2^***^	10.5 ± 4.3	12.0 ± 5.0^#^	97.61	< 0.001
Physical abuse	5.4 ± 1.0^***^	6.6 ± 2.6	7.6 ± 4.3	23.06	< 0.001
Sexual abuse	5.4 ± 0.9^***^	5.9 ± 2.1	6.4 ± 2.3^#^	16.36	< 0.001
Emotional neglect	8.1 ± 3.6^***^	16.5 ± 5.0	17.3 ± 5.7	119.06	< 0.001
Physical neglect	6.6 ± 1.8^***^	10.1 ± 3.0	10.6 ± 3.7	82.05	< 0.001
**EPI**					
Psychoticism	47.1 ± 8.6^***^	54.9 ± 11.2	56.5 ± 10.9	37.84	< 0.001
Extraversion	51.2 ± 12.1^***^	32.8 ± 9.9	37.2 ± 12.1^##^	87.88	< 0.001
Neuroticism	48.1 ± 9.7^***^	68.7 ± 6.8	70.3 ± 6.1^#^	153.27	< 0.001
Lie	47.1 ± 9.4	45.8 ± 9.5	44.1 ± 9.9	5.43	0.07
**Categorical variables (%)**					
Suffering childhood trauma	11 (13.9%)^***^	101 (77.1%)	88 (84.6%)	114.49	< 0.001
Suffering emotional abuse	0 (0%)^***^	40 (30.5%)	43 (41.3%)^#^	41.41	< 0.001
Suffering physical abuse	1 (1.3%)^***^	17 (13.0%)	22 (21.2%)	15.99	< 0.001
Suffering sexual abuse	2 (2.5%)^***^	15 (11.5%)	21 (20.2%)	13.25	0.001
Suffering emotional neglect	5 (6.3%)^***^	87 (66.4%)	72 (69.2%)	89.32	< 0.001
Suffering physical neglect	8 (10.1%)^***^	78 (59.5%)	61 (58.7%)	57.09	< 0.001
Sex (female)	55 (67.9%)	93 (71.0%)	84 (80.8%)	4.06	0.131
Family history	0 (0.0%)^***^	27 (20.6%)	18 (17.3%)	15.82	< 0.001

*Post-hoc* analysis significance is reported as follows:

^*^p < 0.05, ^***^p < 0.001 mood disorder vs. health control (HC).

^#^p < 0.05, ^##^p < 0.01, ^###^p < 0.001 Unipolar Depression (UD) vs. Bipolar Depression (BD).

The difference between the two groups (HC vs. UD and BD groups) showed that the BDI score, BAI score, total CTQ-SF score plus subscale score, and the EPI neuroticism score and psychoticism score, in the UD and BD groups were significantly higher than the HC group (*P* < 0.05). While, the HC group presented with higher scores on the extraversion personality. The difference between the two groups (BD vs. UD) showed that patients with BD were more likely to suffer from higher scores on childhood trauma. Inclusive of the total score of the CTQ (53.9 ± 15.7 vs. 49.6 ± 12.1, *P* < 0.05), subscales of emotional abuse (12.0 ± 5.0 vs. 10.5 ± 4.3, *P* < 0.05), and sexual abuse (6.4 ± 2.3 vs. 5.9 ± 2.0, *P* < 0.05). Furthermore, patients with BD presented with more severe anxiety symptoms (27.4 ± 11.2 vs. 22.1 ± 10.8, *P* < 0.001), and the results of EPI showed that the BD group presented with higher scores on the extraversion and neuroticism personality traits than the UD. And the neuroticism personality of BD is highest among the three groups. There was no significant difference in the remaining subscales (*P* > 0.05; see Table 1).

A binary logistic regression model was established with the diagnosis of mood disorder as an outcome. The model revealed that higher scores of emotional abuse [odds ratio (OR) = 1.47, 95% CI = 1.08–2.01)] and emotional neglect (OR = 1.24, 95% CI = 1.05–1.46), as well as neuroticism (OR = 1.25, 95% CI = 1.16–1.35), were all risk factors associated with mood related depression. Higher scores in extraversion (OR = 0.95, 95% CI = 0.90–0.99) was considered a personality trait with protective factors associated with mood related depression (see Table 2). Similarly, with the diagnosis of BD as an outcome, another binary logistic regression model was established and revealed that **a** higher extraversion score (OR = 1.05, 95% CI = 1.02–1.08) and higher anxiety scores (OR = 1.06, 95% CI = 1.03–1.09) were the risk factors associated with BD (see Table 3).

**Table 2:** Logistics regression analysis between health control and mood disorder group.

	**OR**	**95% confidence interval**		***df***	**Wald**	***P***
		**Lower limit**	**Upper limit**			
**Emotional abuse**	**1.47**	**1.08**	**2.01**	**1**	**5.856**	**0.016**
Physical abuse	1.24	0.70	2.20	1	0.546	0.460
Sexual abuse	0.97	0.49	1.93	1	0.006	0.937
**Emotional neglect**	**1.24**	**1.05**	**1.46**	**1**	**6.244**	**0.012**
Physical neglect	1.13	0.82	1.54	1	0.557	0.455
Psychoticism	1.01	0.95	1.08	1	0.075	0.784
**Extraversion**	**0.95**	**0.90**	**0.99**	**1**	**4.772**	**0.029**
**Neuroticism**	**1.25**	**1.16**	**1.35**	**1**	**31.489**	**0.000**

0 = health control; 1 = mood disorder group. Bold values indicate the variables were statistically significant (*p* < 0.05).

**Table 3:** Logistics regression analysis between Unipolar Depression and Bipolar Depression.

	**OR**	**95% confidence interval**		***df***	**Wald**	***P***
		**Lower limit**	**Upper limit**			
**Extraversion**	**1.05**	**1.02**	**1.08**	**1**	**13.86**	***P*** **<** **0.01**
Total score of CTQ	1.02	0.99	1.04	1	1.91	0.167
Depression	1.01	0.95	1.07	1	0.09	0.770
**Anxiety**	**1.06**	**1.03**	**1.09**	**1**	**16.84**	***P*** **<** **0.01**

0 = Unipolar Depression; 1 = Bipolar Depression. Bold values indicate the variables were statistically significant (*p* < 0.05).

**Figure 2:**
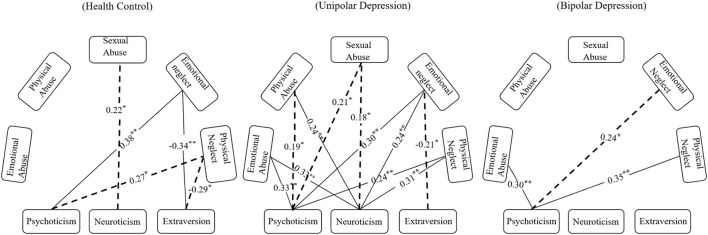
Spearman's correlation analysis of childhood trauma and personality among the different groups. The line represents the correlation between childhood trauma and personality surviving the threshold we set for statistical significance, the dotted line indicates the 0.05 significance levels (*P* < 0.05) and the solid line indicates the 0.01 significance levels (*P* < 0.01).

Spearman's correlation analysis revealed that the extraversion personality trait was negatively associated with childhood trauma. However, neuroticism and psychoticism personality traits were positively associated with childhood trauma. Notably, there was no significant correlation between childhood trauma and neuroticism or psychoticism personality traits in BD (see Figure 2).

## 4. Discussion

In this cross-sectional study of 235 young adult with mood depression, the researchers have assessed childhood trauma rates and personality traits of two groups of patients (UD and BD) and compared them with HC. As our results show, depression in young adults display obvious clinical symptoms, which are manifested in the high depression scores (20.3 vs. 3.0, *P* < 0.001) and high anxiety scores (24.4 vs. 3.4, *P* < 0.001), which was more likely to be found in depressed young adults who had experienced more childhood trauma than the HC (BD vs. UD vs. HC: 84.6, 77.1, 13.9%, respectively). The prevalence of childhood trauma in our sample is mostly aligned with previous work from census surveys like the Netherlands Study of Depression and Anxiety, which reports point prevalence in Dutch individuals, where those who self-report childhood trauma have either a current or remitted mood disorder (88.6%) (27). Similarly, Etain et al. (28) found that BD patients displayed more complex childhood trauma than controls (63 vs. 33%). In terms of those who attended the BD Research Clinic of the New York-Presbyterian Hospital, Garno et al. (29) further found the percentage of emotional abuse was 37%, physical abuse was 24%, sexual abuse was 21%, emotional neglect was 24%, and physical neglect was 12% (29). The results from present research revealed that the most common forms of childhood trauma among the BD group were emotional neglect (69.2%) and physical neglect (58.7%), followed by emotional abuse (41.3%), physical abuse (21.2%), and sexual abuse (20.2%). Comparing the results of the two studies, it can be found that in the former abuse is more serious than neglect, and in the latter it is the complete opposite, which can be attributed to the different outcomes of the population, which is a phenomenon well-worth studying.

There was a statistically significant association between most forms of childhood trauma, especially emotional abuse, emotional neglect, physical abuse, and physical neglect with relation to depression in present study. The association of depression with childhood trauma is consistent with findings from other studies, where childhood trauma increases the risk for depression. Childhood trauma is linked to more severe impairment in individuals with depression (30), for instance, childhood trauma negatively affects the subjective perception of repetitive transcranial magnetic stimulation (rTMS) to the left dorsal lateral prefrontal cortex (LDLPFC) in treatment outcomes for female MDD patients (31). Childhood trauma (in particular childhood physical neglect) also has been reported as having a strong association with suicidal behavior in Treatment-Resistant Depression (32). Studies have explored the role childhood trauma plays in the onset of depression through mild stress system dysregulation [HPA axis (33) and inflammation (34)], brain alterations (reduced mPFC volume and increased amygdala reactivity) (35), and maladaptive personality traits (higher neuroticism) (36). The impact of childhood trauma and personality on the development of depression is complex, research has shown that childhood trauma and neuroticism are positively correlated with depressive scores. In addition, childhood trauma is positively correlated with neuroticism (37). Having a higher score on the personality trait of neuroticism is associated with a higher rate of depression severity (38). Neuroticism is also suggested to be a mediating factor in the relationship between childhood trauma and depression (39).

Another important finding of this study is that a high score on the extraversion personality traits and anxiety symptoms are risk factors for patients in a current depressive episode that result in a diagnosis of BD. Previous studies show that, when compared to UD patients, BD patients have shown lower, higher, or equal neuroticism scores, and higher or equal extraversion scores. The reason for this disunity may be that personality has a certain degree of heredity. In support, a twin study implied that the quality of personality traits (extraversion) can be influenced by heredity (15). Extraversion personality trait is the other important finding of our study, which might be useful in distinguishing UD from BD. Epidemiological and clinical studies have reported a high prevalence of anxiety symptoms in BD patients (40). There is evidence that almost 50% of patients with BD have a comorbid anxiety disorder, and this is tightly associated with morbidity and mortality (41). The etiological research into this co-occurrence is sparse and fails to explain the comorbidity relationships adequately (42), future research should therefore aim to address the comorbidity of BD and anxiety, and longitudinal data is required to understand the relationship of symptoms during the diagnostic journey.

Finally, the results of the correlation between childhood trauma and personality traits show that childhood trauma was positively correlated with neuroticism and psychoticism personality traits, and negatively correlated with extraversion among the total participating young adult. In the BD group participants self-reported more severe childhood trauma, but it seems that the BD's personality is not closely related to childhood trauma like UD and HC. The correlation indicated that the relationship between childhood trauma and personality traits in the BD group was not a simple linear correlation. Our research suggests that the etiological factor and pathogenesis of UD show a preference for environmental factors, such as childhood trauma, while the etiological factor and pathogenesis of UD show a preference for genetic factors, with 30–40% of occurrences of BD involving a genetic factor, according to epidemiological research (43). Understanding heredity within individual differences between BD, UD, and an HC are points of further inquiry.

The strengths of this study included a large sample of medication-naive young adults in a current depressive episode. The results suggested that high scores on extraversion and anxiety were the risk factors for developing BD. This study explored the relationship between childhood trauma and personality among UD, BD, and HC, and provides some indications for future clinical interventions, which is of significance for the early identification of UD and BD. However, some methodological limitations need to consider when explaining our findings. Firstly, although our study has 3 months to 2 years follow-up, which is just used to determine the participants' diagnosis, the design of this study is still a cross-sectional design. Therefore, a causal relationship between personality traits, mood depression, and associated risk factors could not be generated. Secondly, other potentially associated factors of mood depression, such as residential instability and socioeconomic status were not examined (44). Thirdly, the measurement of childhood trauma and individual personality traits depended on self-report inventories, and the clinical emotional state of the participants was not controlled for when filling out the questionnaire. Whether personality measurement is stable with time or independent of emotional state is a debate about the “state-trait phenomenon,” which has been a long-standing controversy. Therefore, it cannot be predicted how these factors will affect the personality scores and childhood traumatic experiences on self-report in our clinical sample. However, some previous evidence supports the stability and emotional independence of some personality traits. A study of adult samples on 4,999 families with over 20,000 individuals measured over 22 years show a high level of genetic and phenotypic stability of neuroticism (45). Finally, Eysenck's work has been ruled unsafe, but the research community has yet to provide guidance on a more appropriate scale to be used in future research. In summary, although there may be emotional state effects in personality measurement, it is unlikely to fully explain our discovery of different personality traits between BD and UD patients, as both BD and UD patients were in a current first depressive episode.

In summary, childhood trauma, as an environmental stimulus, during the early stages of growth, is related to the susceptibility to developing a mood disorder. Personality traits may play an assumed role in the susceptibility to mood disorders by influencing the individual's response to environmental stimuli and stress. Our findings also indicated that different patterns of personality traits persisted in the subtypes of BD and UD, with BD participants showing higher scores on the extraversion personality trait and anxiety symptoms. The combination of personality traits and childhood trauma can be regarded as individual vulnerability factors for developing mood disorders and other related behavioral problems. Our findings add empirical support to the applicability of personality measurement in the study of mood disorders, especially for patients with BD.

## Data availability statement

The original contributions presented in the study are included in the article/supplementary material, further inquiries can be directed to the corresponding authors.

## Ethics statement

The study approval was granted by the Ethics Committee of the Second Xiangya Hospital of Central South University (Approval No. 2018-007) on December 27th, 2018. Written informed consent to participate in this study was provided by the participants' legal guardian/next of kin.

## Author contributions

JL, JZ, and XCa conceived and designed the study. XCh, PL, HT, and CL participated in the acquisition of data. JL analyzed the data and drafted the manuscript. YW, AW, YT, XW, XCa, and JZ revised the manuscript. All authors read and approved the final manuscript.
